# Identification of biomarkers associated with energy metabolism in hypertrophic cardiomyopathy and exploration of potential mechanisms of roles

**DOI:** 10.3389/fcvm.2025.1546865

**Published:** 2025-04-09

**Authors:** Songyan Cai, Tianying Jin, Mintong Liu, Qingyuan Dai

**Affiliations:** ^1^ Department of Cardiology, First Affiliated Hospital of Kunming Medical University, Kunming, Yunnan, China; ^2^ Department of Physical Examination for Cadres, First Affiliated Hospital of Kunming Medical University, Kunming, Yunnan, China

**Keywords:** hypertrophic cardiomyopathy, energy metabolism, immune infiltration, bioinformatics analysis, biomarkers

## Abstract

**Background:**

In hypertrophic cardiomyopathy (HCM), limited reports exist regarding its association with energy metabolism. Here, biomarkers related to energy metabolism in HCM were identified through bioinformatics analysis.

**Methods:**

HCM transcriptome data were acquired from the GEO (GSE36961) database for comparative analysis in order to identify differentially expressed genes (DEGs). Subsequently, the identified DEGs were intersected with key module genes in Weighted gene co-expression network analysis (WGCNA) and energy metabolism related genes (EMRGs) to identify DE-EMRGs. Then, feature biomarkers were screened using the least absolute shrinkage and selection operator (LASSO) regression and support vector machine-recursive feature elimination (SVM-RFE) methods, and the intersection of the feature biomarkers obtained from both methods was used for subsequent analysis. Furthermore, biomarkers defined as biomarkers with consistent expression trends across both GSE36961 and GSE89714 datasets and significant inter-cohort differences were selected for subsequent analysis. Subsequently, an immune analysis was conducted. Additionally, the transcription factors (TFs), and drugs regulating the biomarkers were predicted based on online databases.

**Results:**

The co-selection of seven potential biomarkers based on machine learning identified IGFBP3 and JAK2 as biomarkers in HCM. Upregulation of IGFBP3 and JAK2 in the HCM cohort was observed in the GSE36961 and GSE89714 datasets. Utilizing ssGSEA, it was unveiled that the HCM cohort exhibited elevated ratings of effector memory CD4T cells while displaying diminished scores across 22 other immune cell categories. Notably, JAK2 expression exhibited a strong negative correlation with myeloid-derived suppressor cells (MDSCs) infiltration, while IGFBP3 showed no significant associations with immune cell infiltration. Utilizing NetworkAnalyst, miRNAs and TFs regulating biomarkers expression in HCM were predicted, with hsa-mir-16-5p, hsa-mir-147a, hsa-mir-210b-3p, hsa-let-7b-5p, and hsa-mir-34a-5p identified as regulators of both IGFBP3 and JAK2. GATA2 was also found to be a TF regulating the expression of both biomarkers. Furthermore, the potential therapeutic targets of JAK2 and IGFBP3 in HCM were ruxolitinib and celecoxib, respectively.

**Conclusion:**

In conclusion, the identification of IGFBP3 and JAK2 as biomarkers in HCM, highlight promising avenues for further research and treatment development in HCM.

## 1 Introduction

Hypertrophic cardiomyopathy (HCM) is an autosomal dominant cardiovascular disorder that leads to left ventricular hypertrophy, myocardial hypercontractility, decreased compliance, muscle fiber dysfunctions, and fibrosis (1). The data indicate that the incidence of HCM is 1:200 (2) and it is the most prevalent cause of sudden cardiac death(SCD) among adolescents and athletes (3). Up to 60% of adult HCM patients result from mutations in genes encoding myocardial sarcomeric proteins, among which the most prevalent ones are genes encoding the heavy chains of myosin (MYH7) and myosin-binding protein C (MYBPC3) (4–6). Research has confirmed that genetic mutations play a significant role in HCM. However, in approximately 40% of patients with HCM, the causative gene remains to be identified (3). Previously, HCM was regarded as a malignant disease that was almost incurable. However, with the advancement of medical standards and the enhanced cognition of HCM, the mortality rate of HCM has decreased significantly (7, 8). Nevertheless, there remains a considerable demand for the treatment of HCM. Hence, the development of relevant biomarkers for the treatment of HCM is of utmost urgency.

In recent years, the significance of energy metabolism disorders in the pathogenesis of HCM has been emphasized, encompassing the aberrant conversion of myocardial metabolic substrates from fatty acids to glucose, augmented energy requirements, and low myocardial energy utilization efficiency (9–11). The heart exhibits a high level of flexibility in selecting energy substrates, encompassing fatty acids, lactic acid, glucose, ketone bodies, and amino acids. Approximately 60%-90% of normal cardiac is sustained by the oxidation of fatty acids (12). In the HCM model, diminished expression of long-chain fatty acid transporter (CD36) and acyl-CoA dehydrogenase deficiency activity result in decreased uptake and utilization of fatty acids (13, 14). When the heart undergoes pathological hypertrophy, cardiomyocytes experience relative hypoxia, often leading to alterations in their energy metabolism. In comparison to glucose, fatty acid oxidation necessitates more oxygen and Adenosine triphosphate (ATP). Consequently, cardiomyocytes predominantly rely on glycolysis for ATP production to fulfill their energy demands. In addition, studies have shown that insulin resistance is associated with HCM (15), with significant Insulin Resistance (IR) present in HCM patients without significant diabetes and hypertension (16). Currently, despite extensive research into the mechanism of HCM from various perspectives, there remains a lack of systematic studies on energy metabolism-related genes in HCM. Therefore, it is imperative to integrate multi-platform data to identify key energy metabolism genes and their corresponding regulatory factors involved in HCM, and subsequently investigate the expression, function, and molecular mechanism of these biomarkers. This will facilitate the exploration of new therapeutic targets for HCM.

**Figure 1:**
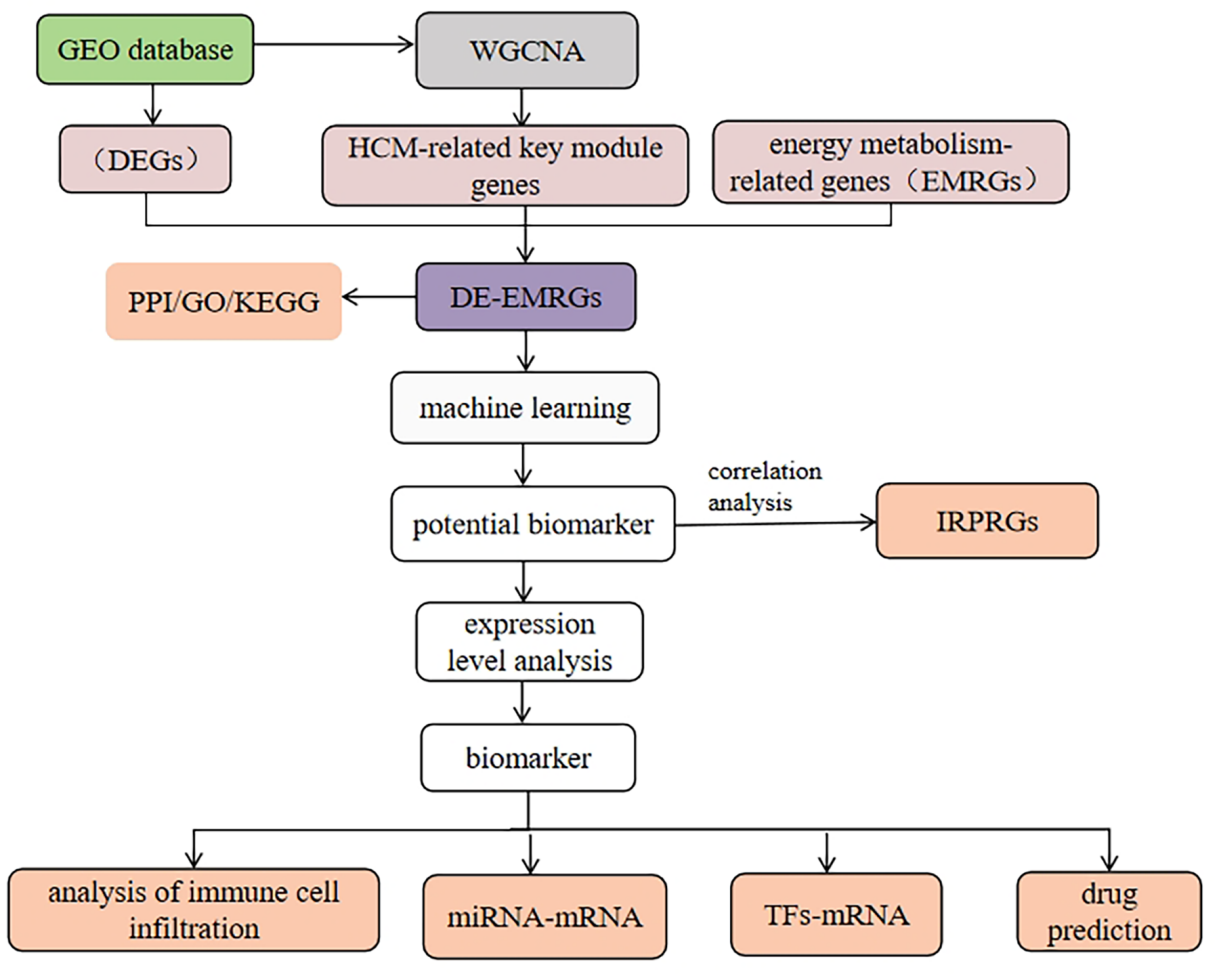
The flow chart of the study design.

This study utilized transcriptome data from HCM patients in the public databases GEO (GSE36961), differentially expression genes (DEGs) were determined in the GSE36961 dataset. Subsequently, the identified DEGs, energy metabolism related genes (EMRGs) and the key module genes were intersected, in order to determine the DE-EMRGs. The DE-EMRGs were screened to acquire biomarkers using machine learning algorithms LASSO, SVM-RFE and expression verification, and explored the biological functions, molecular regulatory networks, and drug prediction of biomarkers, providing new reference for the prevention and treatment of patients with HCM. The analysis flow is shown in Figure 1.

## 2 Materials and methods

### 2.1 Data extraction

Transcriptomic and clinical data were sourced from the GEO database with accessions GSE36961 and GSE89714 at https://www.ncbi.nlm.nih.gov/geo/. The GSE36961 dataset (platform: GPL15389), consisting of heart tissue samples from 106 individuals with HCM and 39 control controls, was utilized for tasks including WGCNA network construction, biomarkers identification, and immunization analyses. Validation of biomarkers expressions was carried out using the GSE89714 dataset (platform: GPL11154), which comprised heart tissues from 5 HCM patients and 4 control individuals. Additionally, we obtained 927 EMRGs from the GeneCards database (https://www.genecards.org/, Version 5.11) by setting the filter condition as Category = Protein Coding, Relevance score ≥ 7 (Supplementary Table S1).

### 2.2 WGCNA

The gene-expression patterns from the GSE36961 dataset were utilized to investigate the HCM-associated module by leveraging the “WGCNA” R package (version 1.70-3) (17). Firstly, gene expression values of GSE36961 dataset were filtered in this study by selecting genes with expression values greater than 1 for sample clustering analysis. Subsequently, through sample clustering analysis, outlier samples were identified and removed to ensure the accuracy of subsequent analytical procedures. Then, an adjacency matrix was developed to delineate the relationship intensity among the nodes according to the adjacency matrix formula (18):

$$s_{ij}=|cor(x_{i},x_{j})|a_{ij}=s_{ij}\beta$$

Within this investigation, the symbols i and j represent distinct genes, while xi and xj indicate expression levels. S_ij_ signifies the correlation coefficient, with a_ij_ denoting the intensity of the correlation between i and j. For this analysis, we establish the adjacency matrix using an optimal soft-threshold power and a scale-free topological index (R^2^) of 0.85. This matrix is subsequently transformed into a topological overlap matrix. The formation of hierarchical clustering trees with modules is achieved through the dynamic cutting of trees (with a module size of 200) to pinpoint key modules by aggregating genes with analogous expression tendencies into the same module. Modules with significant correlation with HCM traits were selected as key modules [|Correlation (cor)| > 0.3, *p*-value < 0.05].

### 2.3 Identification of DE-EMRGs

In the GSE36961 dataset, the DEGs were identified through the application of the “limma” R package (version 3.46.0) (19) (*p*-value < 0.05). For visualization, “ggplot2” R package (v 3.3.6) (PMID: 35751589) and “pheatmap” R package (v 1.0.12) (PMID: 34864868) were utilized to plot the volcano and heatmap, respectively. Subsequently, the identified DEGs were intersected with the EMRGs and key module genes in the WGCNA using the “VennDiagram” R package (version 1.6.20) (20), in order to determine the DE-EMRGs.

### 2.4 Function analysis

To investigate potential interactions among DE-EMRGs in the GSE36961 dataset, the STRING (https://string-db.org) platform was utilized to construct a protein-protein interaction (PPI) network (confidence score > 0.4). Subsequent to this, Gene Ontology (GO) functional and Kyoto Encyclopedia of Genes and Genomes (KEGG) analyses for the DE-EMRGs with verified interactions were conducted employing the “clusterProfiler” R package (21).

### 2.5 Biomarkers screening and validation

Upon obtaining the identified DE-EMRGs as mentioned earlier, two distinct machine learning algorithms were utilized to refine the selection of potential biomarkers. The Least Absolute Shrinkage and Selection Operator (LASSO) was applied through the utilization of the glmnet package (version 4.1-1) (22) to reduce data dimensionality for feature biomarkers selection. Concurrently, a Support Vector Machine Recursive Feature Elimination (SVM-RFE) model was established utilizing the caret package (version 6.0–86, https://CRAN.R-project.org/package = caret) to identify feature biomarkers with the lowest error rate and highest precision. The results obtained by the two algorithms were intersected to produce potential biomarkers, which were displayed in Venn diagram. Subsequently, KEGG enrichment results showed that the insulin resistance pathway was significantly enriched too. Therefore, correlation analysis of potential biomarkers with insulin resistance pathway related genes (IRPRGs) was performed as well as plotting visualisations using ggplot2 (version 3.3.3) (PMID:35751589) (|cor| > 0.3, *p*-value < 0.05). Finally, expression validation was carried out in the GSE36961 and GSE89714 datasets, with potential biomarkers showing consistent expression trends in both datasets and significant inter-cohort differences being defined as biomarkers.

### 2.6 Analysis of immune correlation

The infiltration levels in the GSE36961 dataset were quantified using ssGSEA (23), and intergroup differences were examined. Additionally, Spearman's rank correlation analysis was employed to assess the association of biomarkers with immune cell (|cor| > 0.3, *p*-value < 0.05).

### 2.7 Multifactorial regulatory network construction of biomarkers and prediction of potential therapeutic agents

To identify miRNAs and TFs, the TarBase v8.0 database and JASPAR database on the NetworkAnalyst platform (https://www.networkanalyst.ca/) were utilized for prediction. Subsequently, the DGIdb website (https://dgidb.genome.wustl.edu) was employed to predict target drugs for biomarkers with an interaction_cohort_score ≥ 0.2, aiming to identify potential therapeutic small molecule compounds for HCM patients. Additionally, networks involving miRNA-mRNA interactions, TFs-mRNA interactions, and drug-biomarkers were established utilizing the “Cytoscape” R package (version 3.8.2) (24).

### 2.8 Statistical analysis

All statistical analyses and visual plotting of the results were performed based on R software (https://www.r-project.org/, version 4.0.3, R Statistical Computing Project). The wilcox test was used to compare the ratio of immune cells between HCM and control samples, and correlation analysis of biomarkers with immune cell by using spearman coefficient.

## 3 Results

### 3.1 Identification of the HCM-related modules and genes through WGCNA

**Figure 2:**
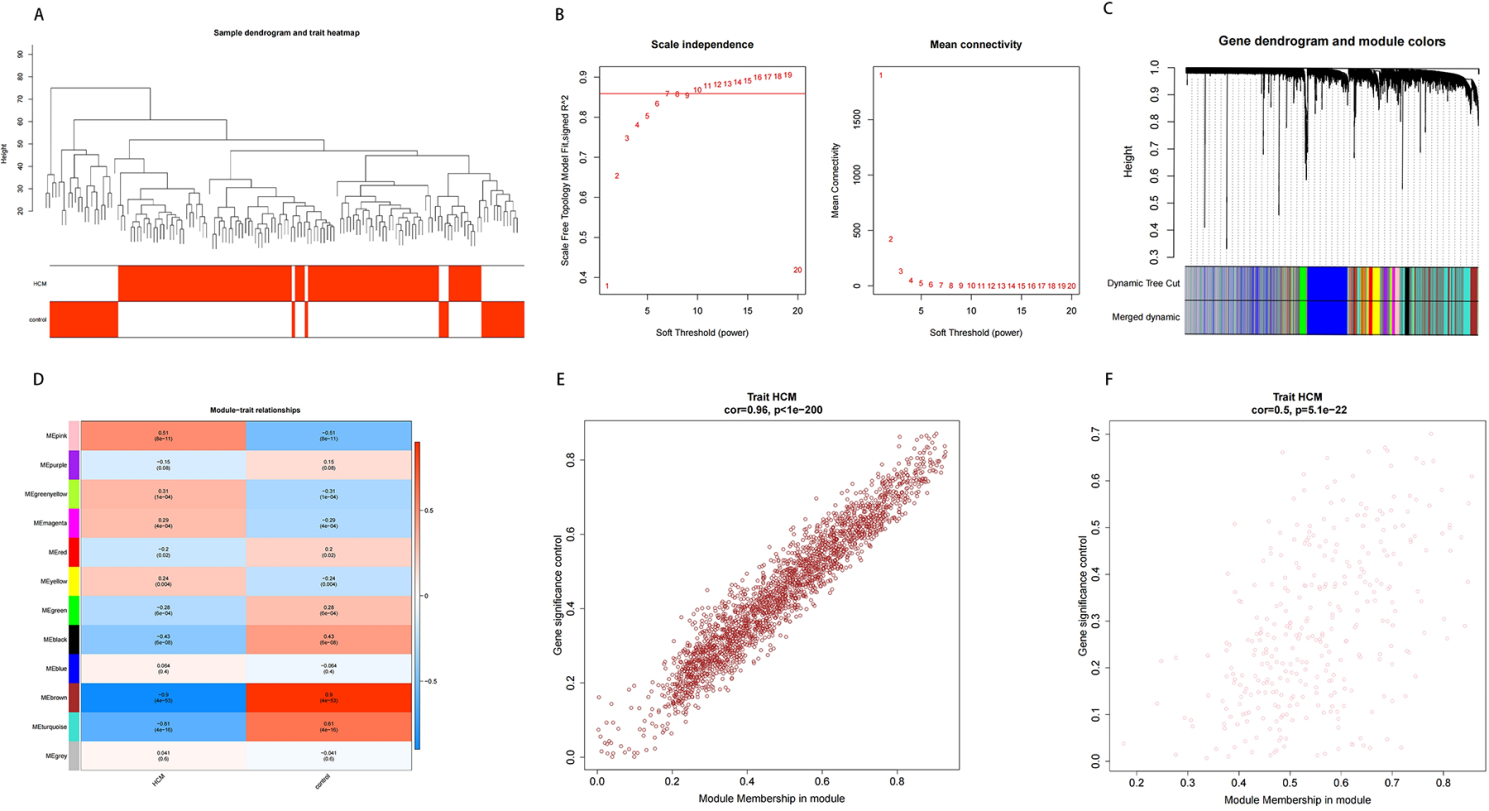
Results of WGCNA. **(A)** Sample clustering and phenotypic heat maps. The branches represent the samples and the ordinate represents the height of the hierarchical clustering. Branch corresponding red clinical character represents the sample belong to such properties. **(B)** Soft threshold filtering. The horizontal axis represents the power value of the weight parameter; the vertical axis of the left figure is scale-free fit index (signed R2); the higher the square of the correlation coefficient, the closer the network is to the scale-free distribution; the vertical axis of the right figure represents the mean value of all gene adjacency functions in the corresponding gene module. **(C,D)** Dynamic tree cutting before and after module mergin and correlation heat map of modules and HCM. **(E,F)** Correlation between module gene and HCM.

To uncover modules and genes associated with HCM, WGCNA was utilized to construct a co-expression network utilizing all samples and genes present in the dataset. Sample dendrogram as well as HCM and control heatmap were mapped (Figure 2A). Then, a scaleless network was constructed with the optimal soft-threshold power (*β*) was set as 7 and the index of scale-free topologies was set as 0.85 (Figure 2B). A hierarchical clustering tree with modules was formed by introducing genes with similar expression patterns into the same module by a dynamic tree-cutting (module size = 200), and 12 modules were identified (Figures 2C,D). Among 12 modules, MEpink (cor = 0.51, *p*-value = 8e-11) and MEbrown (cor = 0.96, *p*-value = 4e-53) had the highest correlation with HCM (Figures 2E,F). Therefore, these two modules and 2,710 genes in these two modules were finally used for the subsequent analysis.

### 3.2 Identification of DE-EMRGs

**Figure 3:**
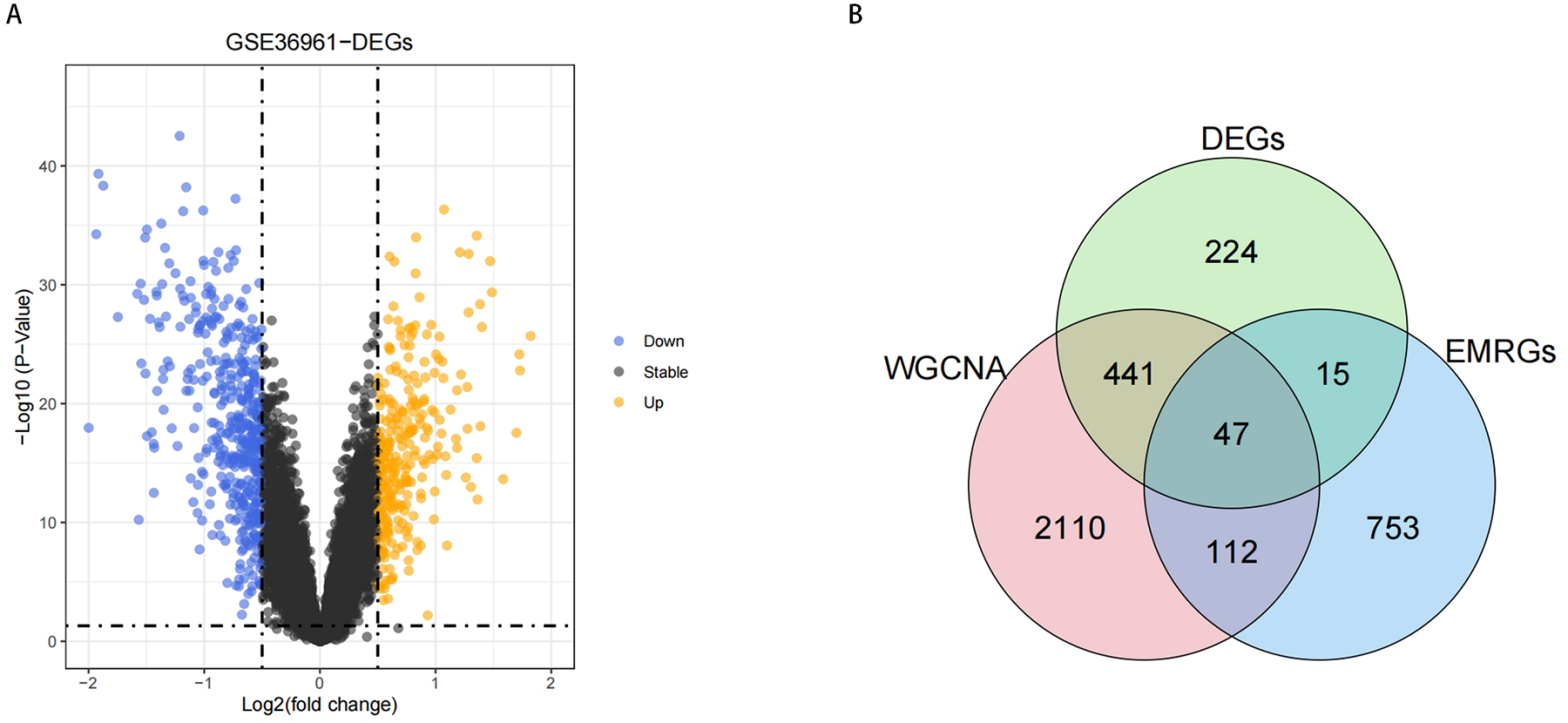
Results of DE-EMRGs. **(A)** Volcano plot depicting differentially expressed genes between HCM and control samples in the GSE36961 dataset. The Orange dots represent significantly upregulated genes, while the Purple dots represent significantly downregulated genes. **(B)** Venn diagram of DE-EMRGs in HCM.

To identify DE-EMRGs in HCM, we initially isolated DEGs from HCM and control samples within the GSE36961 dataset. As illustrated in Figure 3A, we discovered a sum of 727 DEGs, with 288 genes showing reduced expression and 439 genes exhibiting increased expression in HCM samples. Subsequently, we derived 47 DE-EMRGs for further analysis by intersecting the DEGs, key module genes, and EMRGs (Figure 3B, Supplementary Table S2).

### 3.3 A PPI network of DE-EMRGs and functional analysis

**Figure 4:**
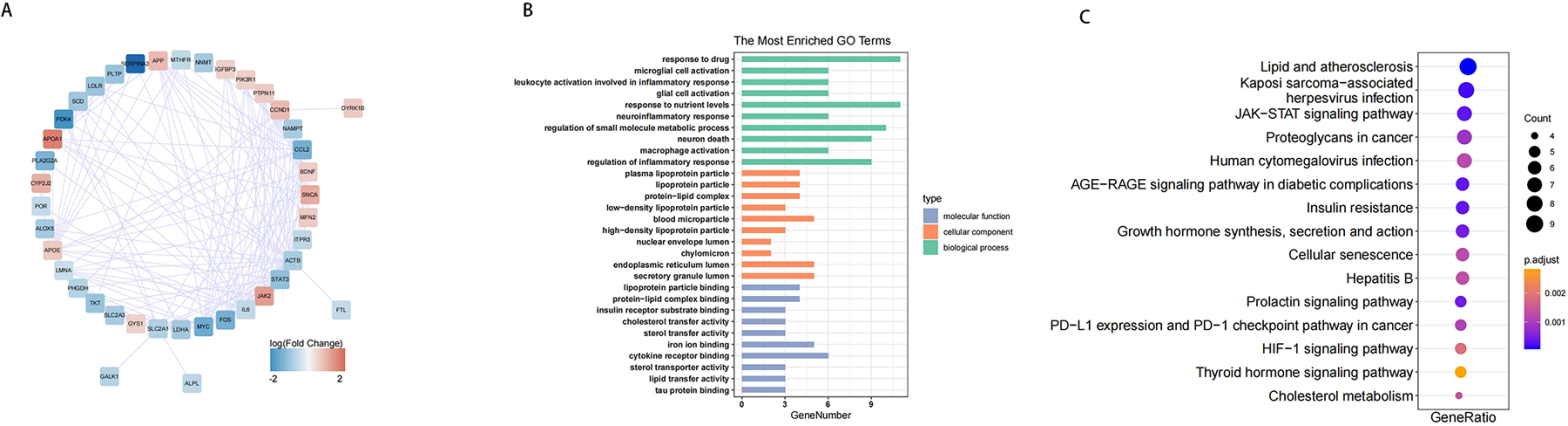
PPI network of DE-EMRGs and functional analysis. **(A)** protein-protein interaction network of DE-EMRGs. **(B)** GO enrichment bar chart of DE-EMRGs. **(C)** KEGG-enriched bubble map of DE-EMRGs.

To investigate the interactions among the 47 DE-EMRGs, a protein-protein interaction (PPI) network was constructed. This resulted in a PPI network comprising 170 interactions and 41 nodes. Therefore, 41 from the 47 DE-EMGRs were contained in the final PPI network and used for subsequent analysis (Figure 4A, Supplementary Table S3). Subsequently, GO and KEGG analyses were performed to investigate the role of the 41 DE-EMRGs in various biological processes. The GO indicated that these DE-EMRGs were predominantly associated with ten terms, including response to drug, microglial cell activation, and response to nutrient in biological process; these DE-EMRGs were predominantly associated with ten categories, including plasma lipoprotein particle, lipoprotein particle, and blood microparticle in cellular component; these DE-EMRGs were mainly involved in ten terms such as iron ion binding, tau protein binding, and sterol transfer activity in molecular function (Figure 4B). In KEGG terms, these DE-EMRGs were significantly associated with cholesterol metabolism, thyroid hormone signaling, and insulin resistance, etc. pathway (Figure 4C).

### 3.4 Potential biomarkers were selected and correlation analysis

**Figure 5:**
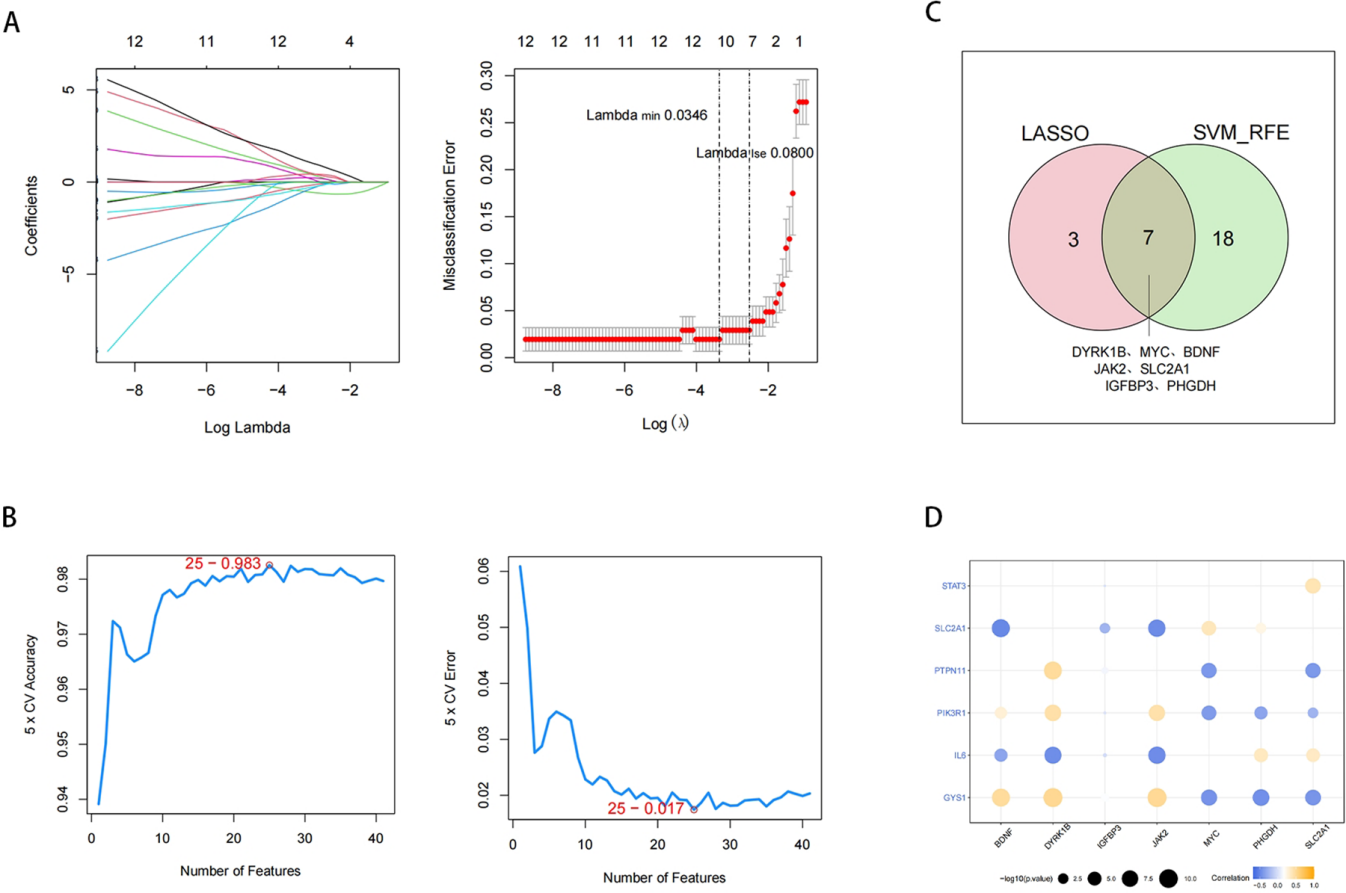
Identification of potential biomarkers. **(A)** Feature biomarkers were screened by LASSO regression analysis. The horizontal axis deviance represents the proportion of residual explained by the model, showing the relationship between the number of feature biomarkers and the proportion of residual explained (dev), and the vertical axis is the coefficient of feature biomarkers (left); The horizontal axis is log(Lambda), and the vertical axis represents the error of cross-validation (right). **(B)** SVM feature number and error rate and accuracy rate. **(C)** Venn diagram of LASSO and SVM-REF analysis. **(D)** Circle diagram of potential biomarkers correlating with IRPRGs.

We performed the LASSO (lambda min=0.0346) to identify 10 feature biomarkers (DYRK1B, SERPINA3, MYC, BDNF, JAK2, SLC2A1, IGFBP3, PHGDH, PTPN11, and CCND1) (Figure 5A). Meanwhile, the SVM-RFE approach was applied to select a set of 25 feature biomarkers (JAK2, IGFBP3, MYC, LMNA, PDK4, DYRK1B, MTHFR, FTL, BDNF, CYP2J2, SLC2A1, GALK1, PLA2G2A, ALOX5, CCL2, IL6, NNMT, LDHA, ALPL, APOE, GYS1, NAMPT, ITPR3, PHGDH, and FOS) (Figures 5B). Subsequently, a total of 28 DE-EMRGs were identified by combining the DE-EMRGs identified through the aforementioned approach, of which 7 potential biomarkers (DYRK1B, MYC, BDNF, JAK2, SLC2A1, IGFBP3, and PHGDH) were selected simultaneously by both methods (Figure 5C). Finally, correlation analysis of potential biomarkers with IRPRGs revealed strong positive/negative correlations between potential biomarkers and IRPRGs, such as JAK2 has a positive correlation with GYS1 and negatively correlated with SLC2A1. However,IGFBP3 shows little correlations with IRPRGs (Figure 5D, Supplementary Table S4).

### 3.5 IGFBP3 and JAK2 were identified biomarkers

**Figure 6:**
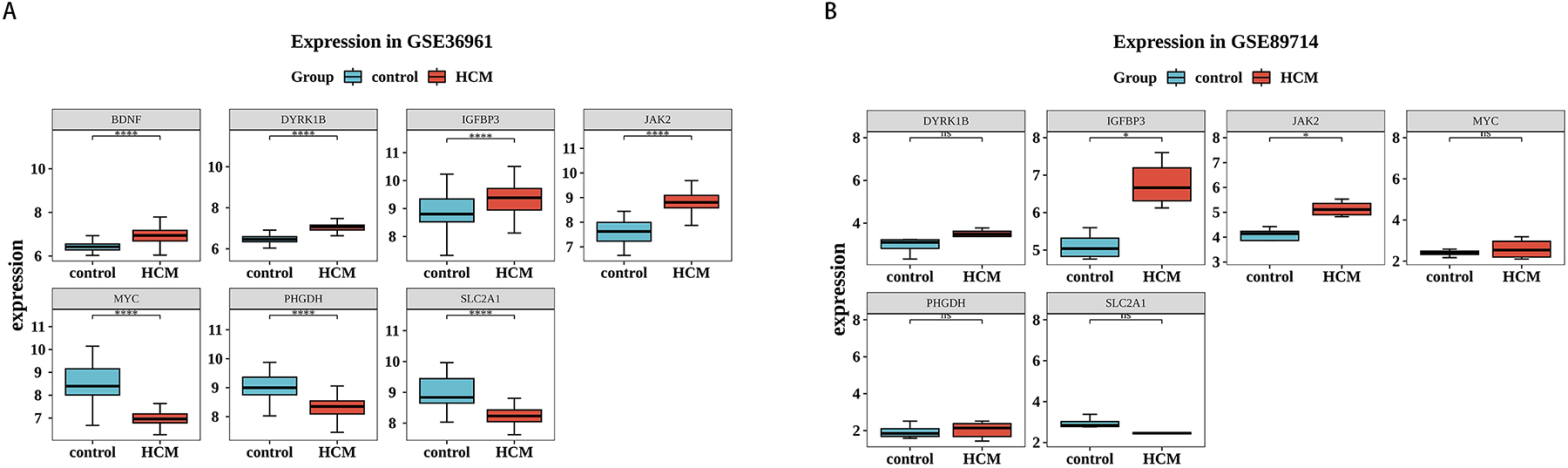
Validation of biomarkers. **(A,B)** Expression of biomarkers in HCM and control samples in the training set (left) and validation set (right). Red is the HCM sample, and blue-green is the control sample.

To gain deeper insights into the expression patterns of the 7 potential biomarkers within the context of the disease, the expression profiles of the 7 potential biomarkers in the HCM and control cohorts were demonstrated in the GSE36961 and GSE89714 datasets. Among them, 2 biomarkers (IGFBP3 and JAK2) exhibited consistent expression trends in both the GSE36961 and GSE89714 datasets, showing significantly higher expression in the HCM cohort (Figure 6A,B). Consequently, IGFBP3 and JAK2 were chosen as biomarkers for further examination.

### 3.6 Biomarkers were associated with immunity

**Figure 7:**
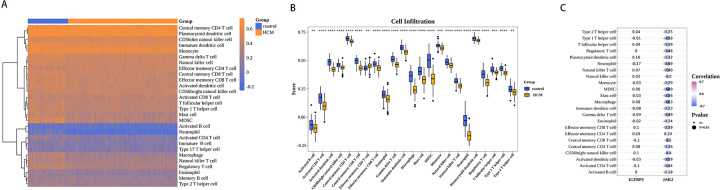
Biomarkers were associated with immunity. **(A)** Immune cell scores of HCM and control samples. **(B)** Differences in HCM and control samples infiltrated immune cells. **(C)** Heat map of correlation between biomarkers and differential immune cells. Purple represents a positive correlation, blue represents a negative correlation, and darker colors represent higher correlations. Dot size indicates significance.

The proportion of the 28 immune cell types evaluated by ssGSEA in each sample is depicted in a heatmap (Figure 7A). Significant differences in the scores of 23 immune cells were noted across the cohorts. In the HCM cohort, effector memory CD4T cells had higher scores, whereas the scores of the remaining 22 immune cells were lower. These included eosinophils, mast cells, and monocytes (Figure 7B). Subsequently, spearman correlation analysis was performed to examine the association of biomarkers with immune cells. The expression of JAK2 was generally inversely correlated with the infiltration of various immune cells, showing the strongest negative correlation with myeloid-derived suppressor cells (MDSCs) (cor = −0.69, *p*-value < 0.05). In contrast, the relationship of IGFBP3 with the infiltration of different immune cells was not notably significant (Figure 7C, Supplementary Table S5).

### 3.7 Multifactorial regulatory network of biomarkers and prediction of potential therapeutic agents

**Figure 8:**
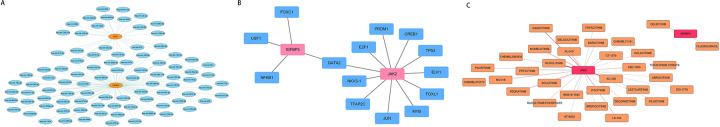
Multifactor regulatory networks and drug prediction of biomarkers. **(A)** miRNA- biomarkers regulatory network. Blue represents miRNA, and yellow indicates the biomarkers. **(B)** TF- biomarkers regulatory network. Blue represents TF, and Pink represents biomarkers. **(C)** Drug- biomarkers relationship network. The pink nodes represent the biomarkers, and the yellow nodes are drugs; a darker line color indicates a higher interaction_group_score.

As shown in Figure 8A, 90 miRNAs were finally predicted (Supplementary Table S6). IGFBP3 and JAK2 were both regulated by hsa-mir-16-5p, hsa-mir-147a, hsa-mir-210b-3p, hsa-let-7b-5p, and hsa-mir-34a-5p, etc. Furthermore, 14 TFs were identified to participate in regulating the expression of the biomarkers., among which GATA2 can simultaneously regulate IGFBP3 and JAK2 (Figure 8B). Next, the DGIdb web server (interaction_cohort_score≥0.2) was utilized to predict targeting agents for biomarkers and to identify small molecule compounds with potential therapeutic effects in HCM patients (Supplementary Table S7). Based on the data presented in Figure 8C, it was observed that ruxolitinib exhibited a high binding affinity towards JAK2, while celecoxib showed strong binding capability to IGFBP3.

## 4 Discussion

In recent years, with the in-depth study of the pathogenesis of HCM, more and more evidence shows that changes in energy metabolism play a key role in the occurrence and development of HCM, but the specific role of EMRGs in the occurrence and development of HCM is still largely unknown. Therefore, systematic analysis of EMRGs in HCM may provide a theoretical basis for exploring the molecular mechanism of HCM. In this study, we identified two biomarkers (IGFBP3 and JAK2) as therapeutic target for HCM.

Through GO and KEGG enrichment analysis, DE-EMRGs were significantly enriched in the inflammatory response. The results of the immune infiltration analysis indicated that 23 types of immune cells were differentially infiltrated in HCM. In recent years, immune cells have been extensively investigated in the context of heart disease. Targeted therapy of specific stages of macrophages can inhibit pathological cardiac hypertrophy (25). Furthermore, the existence of GATA3-positive macrophages adversely influences myocardial remodeling during ischemia or pressure overload, while the absence of these macrophages considerably improves cardiac function (26). In inflammatory cardiomyopathy, the density of mast cells increases, and the release of inflammatory mediators could stimulate the activation of cardiac fibroblasts and enhance collagen synthesis, resulting in cardiac fibrosis (27). Recent studies have manifested that B cells can regulate the composition of the myocardial leucocyte pool as well as growth and contraction, exerting a crucial role in the structure and function of the left ventricle (28). Genetic or induced depletion of eosinophils exacerbates cardiac dysfunction and cardiac fibrosis subsequent to myocardial infarction (29). In cardiomyocytes of both humans and mice, eosinophils are capable of inhibiting cardiomyocyte hypertrophy and death, TGF-*β* signaling in cardiac fibroblasts, and the synthesis of fibrosis proteins (30). This is consistent with our research results. In our study, the number of eosinophils in HCM patients decreased. Additionally, studies have shown that the increase in immune cell infiltration and inflammatory cytokines such as tumor necrosis factor-α (TNF-α) and interleukin-6 (IL-6) in the myocardial tissue of HCM patients may contribute to the development and progression of myocardial fibrosis in HCM (31). However, our findings indicate a reduction in immune cell infiltration in HCM, necessitating further elucidation of the role of inflammation in the pathogenesis of HCM.

The IGFBP superfamily encompasses several proteins, among which are binding proteins featuring high affinity for IGF (IGFBP1 to IGFBP6) and IGFBP-related proteins exhibiting low affinity for IGF (IGFBP- rP1-10), with IGFBP3 being the most abundant (32). The expression of IGFBP3 is augmented in HCM, dilated cardiomyopathy, and ischemic cardiomyopathy (33). In HCM, upregulation of IGFBP3 promotes cardiac tissue fibrosis by elevating mRNA levels of extracellular matrix-related genes (e.g., COL1A2, COL3A1, and MMP9); furthermore, increased IGFBP3 expression recruits immune cell infiltration into cardiac tissue, modulates the immune microenvironment and inflammatory responses, and ultimately contributes to adverse clinical outcomes in HCM patients (34). Prior studies have demonstrated that inhibition of IGFBP3 promotes angiogenesis and mitigates cardiac fibrosis and remodeling in mice with diabetic cardiomyopathy (35). IGFBP3 assumes a significant role in glucose homeostasis and can diminish insulin glucose uptake by reducing insulin-stimulated translocation of glucose transporter −4 to the plasma membrane and threonine phosphorylation of Akt (36). Transgenic mice with overexpression of IGFBP3 exhibited mild insulin resistance, accompanied by elevated levels of plasma leptin, glucose, and insulin (37, 38). In our study, the insulin resistance pathway was conspicuously enriched. The emergence of cardiac insulin resistance and the deterioration of mitochondrial oxidative metabolism constitute early metabolic alterations during the development of cardiac hypertrophy, resulting in energy deficiency and potentially causing hypertrophy to progress to heart failure. Studies have shown the presence of insulin resistance in patients with HCM, so insulin resistance may be related to HCM (16, 39, 40). Furthermore, IGFBP3 plays an important role in lipid metabolism. IGFBP3 inhibits adipocyte differentiation by interfering with peroxisome proliferator-activated receptor gamma (PPARgamma) (41). Overexpression of human IGFBP3 suppressed the expression of adipogenic markers adiponectin and resistin, as well as the accumulation of lipid droplets, by activating Smad signaling in 3T3-L1 cells (42). Hence, IGFBP3 might contribute to the development of HCM by influencing the processes of insulin signaling and fat metabolism.

JAK2 is situated on the short arm of chromosome 9 (9p24) and constitutes an essential member of the JAK family. All three members of the JAK family (JAK1, JAK2, and TYK2) as well as all seven members of the STAT family (STAT1, STAT2, STAT3, STAT4, STAT5A, STAT5B, and STAT6) are expressed in the heart (43). When the cytokine interacts with the receptor situated on the cell membrane, JAK2 kinase is triggered, and subsequently phosphorylates and activates the downstream STAT transcription factor, thereby playing a role in numerous physiological processes such as cell differentiation, proliferation, glycolysis, and inflammation (44, 45). KEGG results indicated that the JAK/STAT signaling pathway was conspicuously enriched. The JAK2-STAT3 signaling pathway exerts a crucial role in myocardial inflammatory damage, ventricular remodeling, and cardiomyocyte hypertrophy, thereby contributing to the progression of heart failure (46, 47). Studies have shown that among 72 HCM patients with no known pathogenic gene mutations, rare JAK2 variants were identified in 9 cases (12.5%) (48). The JAK2 V617F mutant was recognized in a patient suffering from myeloproliferative disorder (MPD) and hypertrophic HCM (49), indicating that HCM is associated with JAK2. In confirmed hypertrophic cardiomyopathy (HCM) without JAK2-V617F mutation, upregulated expression of JAK2 in the global left ventricle (LV) and cardiomyocyte nuclei was observed, along with activation of its downstream target STAT3 (50). The activation of the JAK2/STAT3 signaling pathway was detected in hypertrophic hearts elicited by isoproterenol (51, 52) and the inhibition of the activities of JAK2 and STAT3 mitigated myocardial hypertrophy (53). In terms of energy metabolism, the activation of the JAK2/STAT3 signaling pathway is capable of up-regulating the expression of key enzymes within the glycolysis pathway and the translocation of glucose transporters (54–57), thereby enhancing the glucose utilization. After cardiac injury, the heart tends to rely on glycolysis as an energy source, and Pyruvate kinase M2(PKM2) assumes a significant role in this process. Additionally, myocardial fibrosis is one of the typical pathological alterations of HCM, and PKM2 can also be involved in promoting cardiac fibrosis via mechanisms such as JAK2/STAT3 signal activation (58). The activation of JAK2 is correlated with the emergence of insulin resistance (59–63), and the depletion of JAK2 in adipocytes boosts insulin sensitivity in the liver (64). In adipocytes, the JAK2/STAT3 signaling pathway has the ability to up-regulate the expression of fatty acid synthesis-related genes such as Fatty acid synthase (FASN) and enhance the synthesis of fatty acids, thereby influencing the balance of cellular energy metabolism (65). In conclusion, JAK2 might play a crucial role in HCM via its influence over energy metabolism.

This research utilizes the relevant software and database for the prediction of microRNAs, transcription factors, and drugs based on the target gene. MicroRNAs and transcription factors play a crucial role in regulating gene expression, such as leading to the occurrence of tumors, metastasis, resistance, etc (66). Through the construction of the miRNA-mRNA network, it was discovered that IGFBP3 and JAK2 were regulated by hsa-mir-16-5p, hsa-mir-147a, hsa-mir-200b-3p, hsa-let-7b-5p, and hsa-mir-34a-5p. Moreover, GATA2 is capable of regulating both IGFBP3 and JAK2. Previous research has demonstrated that mutations in GATA2 are correlated with HCM (67). Therefore, the further exploration of microRNAs and transcription factors for understanding the pathogenesis and treatment of HCM holds great significance. Ruxolitinib and celecoxib were respectively predicted to be the target drugs for JAK2 and IGFBP3. Ruxolitinib as JAK1 and selectivity of JAK2 inhibitors, has been the United States Food and Drug Administration (FDA) approved for the treatment of b myelofibrosis (68). In the rabbit model of atherosclerosis, ruxolitinib exhibits efficacy in attenuating the development of aortic atherosclerotic plaque, lowering plasma levels of triglycerides (TG), total cholesterol (TC) and low-density lipoprotein (LDL), while concurrently elevating high-density lipoprotein cholesterol (HDL-C) levels (69). Celecoxib belongs to the nonsteroidal anti-inflammatory drugs (NSAIDs)and has been studied extensively in inflammatory diseases and cancer (70). In cardiovascular disease, celecoxib may be by Notch1/Hes1 signaling pathway to protect the heart from hypertrophy and inflammation (71). In summary, ruxolitinib and celecoxib can be potential drugs for the treatment of HCM, but more therapeutic agents need to be continuously explored.

## 5 Limitation

This study has certain limitations. First, this research is a bioinformatics analysis relying on the transcriptome profiles of public databases, and the small sample size of the validation dataset may affect the reliability and generalizability of the results. Second, the genes identified in this study have not been further examined, and their pathophysiological impacts across different causal genes and clinical phenotypes of hypertrophic cardiomyopathy remain to be validated. Thus, it is necessary to conduct cell, animal, and clinical studies to verify the expression levels of these biomarkers in hypertrophic cardiomyopathy and to deeply explore the specific underlying mechanisms. Additionally, the work of exploring drug targets based on bioinformatics analysis across multiple database sets and different causal genes still needs to be carried out in depth.

## 6 Conclusion

In summary, 41 DE-EMRGs related to HCM were first obtained through bioinformatics analysis in this study, and functional enrichment analysis showed that DE-EMRGs were related to inflammatory response, insulin resistance pathway, JAK/STAT signaling pathway, and lipid and atherosclerosis signaling pathways. In combination with machine learning algorithms LASSO and SVM-RFE, seven genes were identified as potential biomarkers for HCM, and expression validation identified two biomarkers (JAK2 and IGFBP3). Two regulatory networks of biomarkers (miRNA-mRNA and TFs-mRNA) were constructed, and drug prediction and immune infiltration of biomarkers were performed, providing new insights for HCM treatment and prevention. In the future, it is necessary to conduct further cell experiments, animal experiments and clinical studies to confirm the above conclusions, and finally hope to provide new ideas for clinical diagnosis and treatment of the disease.

## Data Availability

The original contributions presented in the study are included in the article/Supplementary Material, further inquiries can be directed to the corresponding author.

## References

[1] Teekakirikul P Padera RF Seidman JG Seidman CE. Hypertrophic cardiomyopathy: translating cellular cross talk into therapeutics. J Cell Biol. (2012) 199(3):417–21. 10.1083/jcb.201207033 23109667 PMC3483129

[2] Semsarian C Ingles J Maron MS Maron BJ. New perspectives on the prevalence of hypertrophic cardiomyopathy. J Am Coll Cardiol. (2015) 65(12):1249–54. 10.1016/j.jacc.2015.01.019 25814232

[3] Marian AJ Braunwald E. Hypertrophic cardiomyopathy: genetics, pathogenesis, clinical manifestations, diagnosis, and therapy. Circ Res. (2017) 121(7):749–70. 10.1161/CIRCRESAHA.117.311059 28912181 PMC5654557

[4] Goldspink PH Warren CM Kitajewski J Wolska BM Solaro RJ. A perspective on personalized therapies in hypertrophic cardiomyopathy. J Cardiovasc Pharmacol. (2021) 77(3):317–22. 10.1097/FJC.0000000000000968 33298734 PMC7933064

[5] Ito K Patel PN Gorham JM McDonough B DePalma SR Adler EE Identification of pathogenic gene mutations in LMNA and MYBPC3 that alter RNA splicing. Proc Natl Acad Sci U S A. (2017) 114(29):7689–94. 10.1073/pnas.1707741114 28679633 PMC5528995

[6] Millat G Bouvagnet P Chevalier P Dauphin C Jouk PS Da Costa A Prevalence and spectrum of mutations in a cohort of 192 unrelated patients with hypertrophic cardiomyopathy. Eur J Med Genet. (2010) 53(5):261–7. 10.1016/j.ejmg.2010.07.007 20624503

[7] Maron BJ Desai MY Nishimura RA Spirito P Rakowski H Towbin JA Management of hypertrophic cardiomyopathy: JACC state-of-the-art review. J Am Coll Cardiol. (2022) 79(4):390–414. 10.1016/j.jacc.2021.11.021 35086661

[8] Maron BJ Rowin EJ Maron MS. Hypertrophic cardiomyopathy: new concepts and therapies. Annu Rev Med. (2022) 73:363–75. 10.1146/annurev-med-042220-021539 35084989

[9] Nollet EE Schuldt M Sequeira V Binek A Pham TV Schoonvelde SAC Integrating clinical phenotype with multiomics analyses of human cardiac tissue unveils divergent metabolic remodeling in genotype-positive and genotype-negative patients with hypertrophic cardiomyopathy. Circ Genom Precis Med. (2024) 17(3):e004369. 10.1161/CIRCGEN.123.004369 38853772 PMC11188634

[10] Ranjbarvaziri S Kooiker KB Ellenberger M Fajardo G Zhao M Vander Roest AS Altered cardiac energetics and mitochondrial dysfunction in hypertrophic cardiomyopathy. Circulation. (2021) 144(21):1714–31. 10.1161/CIRCULATIONAHA.121.053575 34672721 PMC8608736

[11] Ashrafian H Redwood C Blair E Watkins H. Hypertrophic cardiomyopathy:a paradigm for myocardial energy depletion. Trends Genet. (2003) 19(5):263–8. 10.1016/S0168-9525(03)00081-7 12711218

[12] Ritterhoff J Tian R. Metabolic mechanisms in physiological and pathological cardiac hypertrophy: new paradigms and challenges. Nat Rev Cardiol. (2023) 20(12):812–29. 10.1038/s41569-023-00887-x 37237146

[13] Sacchetto C Sequeira V Bertero E Dudek J Maack C Calore M. Metabolic alterations in inherited cardiomyopathies. J Clin Med. (2019) 8(12):2195. 10.3390/jcm8122195 31842377 PMC6947282

[14] Aoyama T Souri M Ushikubo S Kamijo T Yamaguchi S Kelley RI Purification of human very-long-chain acyl-coenzyme A dehydrogenase and characterization of its deficiency in seven patients. J Clin Invest. (1995) 95(6):2465–73. 10.1172/JCI117947 7769092 PMC295925

[15] Hill MC Kadow ZA Long H Morikawa Y Martin TJ Birks EJ Integrated multi-omic characterization of congenital heart disease. Nature. (2022) 608(7921):181–91. 10.1038/s41586-022-04989-3 35732239 PMC10405779

[16] Murakami K Shigematsu Y Hamada M Higaki J. Insulin resistance in patients with hypertrophic cardiomyopathy. Circ J. (2004) 68(7):650–5. 10.1253/circj.68.650 15226630

[17] Langfelder P Horvath S. WGCNA: an R package for weighted correlation network analysis. BMC Bioinformatics. (2008) 9:559. 10.1186/1471-2105-9-559 19114008 PMC2631488

[18] Tian Z He W Tang J Liao X Yang Q Wu Y Identification of important modules and biomarkers in breast cancer based on WGCNA. Onco Targets Ther. (2020) 13:6805–17. 10.2147/OTT.S258439 32764968 PMC7367932

[19] Ritchie ME Phipson B Wu D Hu Y Law CW Shi W Limma powers differential expression analyses for RNA-Sequencing and microarray studies. Nucleic Acids Res. (2015) 43(7):e47. 10.1093/nar/gkv007 25605792 PMC4402510

[20] Chen H Boutros PC. Venndiagram: a package for the generation of highly-customizable venn and Euler diagrams in R. BMC Bioinformatics. (2011) 12(35). 10.1186/1471-2105-12-35 PMC3041657 21269502

[21] Wu T Hu E Xu S Chen M Guo P Dai Z Clusterprofiler 4.0: a universal enrichment tool for interpreting omics data. Innovation (Camb). (2021) 2(3):100141. 10.1016/j.xinn.2021.100141 34557778 PMC8454663

[22] Friedman J Hastie T Tibshirani R. Regularization paths for generalized linear models via coordinate descent. J Stat Softw. (2010) 33(1):1–22. 10.18637/jss.v033.i01 20808728 PMC2929880

[23] Hänzelmann S Castelo R Guinney J. GSVA: gene set variation analysis for microarray and RNA-Seq data. BMC Bioinformatics. (2013) 14:7. 10.1186/1471-2105-14-7 23323831 PMC3618321

[24] Shannon P Markiel A Ozier O Baliga NS Wang JT Ramage D Cytoscape: a software environment for integrated models of biomolecular interaction networks. Genome Res. (2003) 13(11):2498–504. 10.1101/gr.1239303 14597658 PMC403769

[25] Ren Z Yu P Li D Li Z Liao Y Wang Y Single-cell reconstruction of progression trajectory reveals intervention principles in pathological cardiac hypertrophy. Circulation. (2020) 141(21):1704–19. 10.1161/CIRCULATIONAHA.119.043053 32098504

[26] Yang M Song L Wang L Yukht A Ruther H Li F Deficiency of GATA3-positive macrophages improves cardiac function following myocardial infarction or pressure overload hypertrophy. J Am Coll Cardiol. (2018) 72(8):885–904. 10.1016/j.jacc.2018.05.061 30115228 PMC6145461

[27] Zhang W Chancey AL Tzeng HP Zhou Z Lavine KJ Gao F The development of myocardial fibrosis in transgenic mice with targeted overexpression of tumor necrosis factor requires mast cell-fibroblast interactions. Circulation. (2011) 124(19):2106–16. 10.1161/CIRCULATIONAHA.111.052399 22025605 PMC3217207

[28] Adamo L Rocha-Resende C Lin CY Evans S Williams J Dun H Myocardial B cells are a subset of circulating lymphocytes with delayed transit through the heart. JCI Insight. (2020) 5(3):e134700. 10.1172/jci.insight.134700 31945014 PMC7098796

[29] Liu J Yang C Liu T Deng Z Fang W Zhang X Eosinophils improve cardiac function after myocardial infarction. Nat Commun. (2020) 11(1):6396. 10.1038/s41467-020-19297-5 33328477 PMC7745020

[30] Yang C Li J Deng Z Luo S Liu J Fang W Eosinophils protect pressure overload- and *β*-adrenoreceptor agonist-induced cardiac hypertrophy. Cardiovasc Res. (2023) 119(1):195–212. 10.1093/cvr/cvac060 35394031 PMC10022866

[31] Monda E Palmiero G Rubino M Verrillo F Amodio F Di Fraia F Molecular basis of inflammation in the pathogenesis of cardiomyopathies. Int J Mol Sci. (2020) 21(18):6462. 10.3390/ijms21186462 32899712 PMC7554875

[32] Jin L Shen F Weinfeld M Sergi C. Insulin growth factor binding protein 7 (IGFBP7)-related cancer and IGFBP3 and IGFBP7 crosstalk. Front Oncol. (2020) 10:727. 10.3389/fonc.2020.00727 32500027 PMC7242731

[33] Granata R Broglio F Migliorino D Cutrupi S Baldanzi G Sireno M Neonatal and adult human heart tissues from normal subjects and patients with ischemic, dilated or hypertrophic cardiomyopathy express insulin-like growth factor binding protein-3 (IGFBP-3). J Endocrinol Invest. (2000) 23(11):724–6. 10.1007/BF03345060 11194704

[34] Li Y Zhang W Dai Y Chen K. Identification and verification of IGFBP3 and YTHDC1 as biomarkers associated with immune infiltration and mitophagy in hypertrophic cardiomyopathy. Front Genet. (2022) 13:986995. 10.3389/fgene.2022.986995 36267414 PMC9577180

[35] Li CL Liu B Wang ZY Xie F Qiao W Cheng J Salvianolic acid B improves myocardial function in diabetic cardiomyopathy by suppressing IGFBP3. J Mol Cell Cardiol. (2020) 139:98–112. 10.1016/j.yjmcc.2020.01.009 31982427

[36] Haywood NJ Slater TA Matthews CJ Wheatcroft SB. The insulin like growth factor and binding protein family: novel therapeutic targets in obesity & diabetes. Mol Metab. (2019) 19:86–96. 10.1016/j.molmet.2018.10.008 30392760 PMC6323188

[37] Ruan W Lai M. Insulin-like growth factor binding protein: a possible marker for the metabolic syndrome? Acta Diabetol. (2010) 47(1):5–14. 10.1007/s00592-009-0142-3 19771387

[38] Stanley TL Fourman LT Zheng I McClure CM Feldpausch MN Torriani M Relationship of IGF-1 and IGF-binding proteins to disease severity and glycemia in nonalcoholic fatty liver disease. J Clin Endocrinol Metab. (2021) 106(2):e520–e33. 10.1210/clinem/dgaa792 33125080 PMC7823253

[39] Shigematsu Y Hamada M Nagai T Nishimura K Inoue K Suzuki J Risk for atrial fibrillation in patients with hypertrophic cardiomyopathy: association with insulin resistance. J Cardiol. (2011) 58(1):18–25. 10.1016/j.jjcc.2011.03.001 21515029

[40] Wei Z Zhu E Ren C Dai J Li J Lai Y. Triglyceride-glucose index independently predicts new-onset atrial fibrillation after septal myectomy for hypertrophic obstructive cardiomyopathy beyond the traditional risk factors. Front Cardiovasc Med. (2021) 8:692511. 10.3389/fcvm.2021.692511 34368252 PMC8342798

[41] Chan SS Schedlich LJ Twigg SM Baxter RC. Inhibition of adipocyte differentiation by insulin-like growth factor-binding protein-3. Am J Physiol Endocrinol Metab. (2009) 296(4):E654–63. 10.1152/ajpendo.90846.2008 19141684

[42] de Silva HC Firth SM Twigg SM Baxter RC. Interaction between IGF binding protein-3 and TGF*β* in the regulation of adipocyte differentiation. Endocrinology. (2012) 153(10):4799–807. 10.1210/en.2011-1444 22910030

[43] Xuan YT Guo Y Han H Zhu Y Bolli R. An essential role of the JAK-STAT pathway in ischemic preconditioning. Proc Natl Acad Sci U S A. (2001) 98(16):9050–5. 10.1073/pnas.161283798 11481471 PMC55371

[44] Morris R Kershaw NJ Babon JJ. The molecular details of cytokine signaling via the JAK/STAT pathway. Protein Sci. (2018) 27(12):1984–2009. 10.1002/pro.3519 30267440 PMC6237706

[45] Hu X Li J Fu M Zhao X Wang W. The JAK/STAT signaling pathway: from bench to clinic. Sig Transduct Target Ther. (2021) 6(1):402. 10.1038/s41392-021-00791-1 PMC8617206 34824210

[46] Wagner MA Siddiqui MA. The JAK-STAT pathway in hypertrophic stress signaling and genomic stress response. JAKSTAT. (2012) 1(2):131–41. 10.4161/jkst.20702 24058762 PMC3670293

[47] Terrell AM Crisostomo PR Wairiuko GM Wang M Morrell ED Meldrum DR. Jak/STAT/SOCS signaling circuits and associated cytokine-mediated inflammation and hypertrophy in the heart. Shock. (2006) 26(3):226–34. 10.1097/01.shk.0000226341.32786.b9 16912647

[48] Xu J Liu X Dai Q. Integration of transcriptomic data identifies key hallmark genes in hypertrophic cardiomyopathy. BMC Cardiovasc Disord. (2021) 21(1):330. 10.1186/s12872-021-02147-7 34225646 PMC8259117

[49] Gattenlohner S Ertl G Einsele H Kircher S Muller-Hermelink HK Marx A. Cardiac JAK2 mutation V617F in a patient with cardiomyopathy and myeloproliferative disease. Ann Intern Med. (2008) 149(1):69–71. 10.7326/0003-4819-149-1-200807010-00027 18591647

[50] Maron BA Wang RS Shevtsov S Drakos SG Arons E Wever-Pinzon O Individualized interactomes for network-based precision medicine in hypertrophic cardiomyopathy with implications for other clinical pathophenotypes. Nat Commun. (2021) 12(1):873. 10.1038/s41467-021-21146-y 33558530 PMC7870822

[51] Zhao L Wu D Sang M Xu Y Liu Z Wu Q. Stachydrine ameliorates isoproterenol-induced cardiac hypertrophy and fibrosis by suppressing inflammation and oxidative stress through inhibiting NF-*κ*B and JAK/STAT signaling pathways in rats. Int Immunopharmacol. (2017) 48:102–9. 10.1016/j.intimp.2017.05.002 28499193

[52] Chang L Yang R Wang M Liu J Wang Y Zhang H Angiotensin II type-1 receptor-JAK/STAT pathway mediates the induction of visfatin in angiotensin II-induced cardiomyocyte hypertrophy. Am J Med Sci. (2012) 343(3):220–6. 10.1097/MAJ.0b013e31822993ff 21841463

[53] Al-Rasheed NM Al-Oteibi MM Al-Manee RZ Al-Shareef SA Al-Rasheed NM Hasan IH Simvastatin prevents isoproterenol-induced cardiac hypertrophy through modulation of the JAK/STAT pathway. Drug Des Devel Ther. (2015) 9:3217–29. 10.2147/DDDT.S86431 26150695 PMC4484667

[54] Chen J Gao P Peng L Liu T Wu F Xu K Downregulation of STK25 promotes autophagy via the Janus kinase 2/signal transducer and activator of transcription 3 pathway in colorectal cancer. Mol Carcinog. (2022) 61(6):572–86. 10.1002/mc.23403 35349179

[55] Kitakaze T Jiang H Nomura T Hironao KY Yamashita Y Ashida H. Kaempferol promotes glucose uptake in myotubes through a JAK2-dependent pathway. J Agric Food Chem. (2020) 68(47):13720–9. 10.1021/acs.jafc.0c05236 33197173

[56] Wen XD Zhang YL Yang L Ye Z Fu GC Hu YH Angelica sinensis polysaccharide and Astragalus membranaceus polysaccharide accelerate liver regeneration by enhanced glycolysis via activation of JAK2/STAT3/HK2 pathway. Molecules. (2022) 27(22):7890. 10.3390/molecules27227890 36431990 PMC9695464

[57] Zheng X Gou Y Jiang Z Yang A Yang Z Qin S. Icaritin-Induced FAM99A affects GLUT1-mediated glycolysis via regulating the JAK2/STAT3 pathway in hepatocellular carcinoma. Front Oncol. (2021) 11:740557. 10.3389/fonc.2021.740557 34765550 PMC8576446

[58] Ren J Ren B Fu T Ma Y Tan Y Zhang S Pyruvate kinase M2 sustains cardiac mitochondrial integrity in septic cardiomyopathy by regulating PHB2-dependent mitochondrial biogenesis. Int J Med Sci. (2024) 21(6):983–93. 10.7150/ijms.94577 38774750 PMC11103386

[59] Cheng F Yuan G He J Shao Y Zhang J Guo X. Dysregulation of DPP4 is associated with the AMPK/JAK2/STAT3 pathway in adipocytes under insulin resistance Status and liraglutide intervention. Diabetes, Metab Syndr Obes: Targets Ther. (2019) 12:2635–44. 10.2147/DMSO.S229838 PMC6911808 31849507

[60] Lu L Ye X Yao Q Lu A Zhao Z Ding Y Egr2 enhances insulin resistance via JAK2/STAT3/SOCS-1 pathway in HepG2 cells treated with palmitate. Gen Comp Endocrinol. (2018) 260:25–31. 10.1016/j.ygcen.2017.08.023 28842216

[61] Mo M Pan L Deng L Liang M Xia N Liang Y. Iron overload induces hepatic ferroptosis and insulin resistance by inhibiting the Jak2/stat3/slc7a11 signaling pathway. Cell Biochem Biophys. (2024) 82(3):2079–94. 10.1007/s12013-024-01315-8 38801513

[62] Thirone AC JeBailey L Bilan PJ Klip A. Opposite effect of JAK2 on insulin-dependent activation of mitogen-activated protein kinases and akt in muscle cells: possible target to ameliorate insulin resistance. Diabetes. (2006) 55(4):942–51. 10.2337/diabetes.55.04.06.db05-1265 16567515

[63] Zhang Y Zhou B Deng B Zhang F Wu J Wang Y Amyloid-*β* induces hepatic insulin resistance *in vivo* via JAK2. Diabetes. (2013) 62(4):1159–66. 10.2337/db12-0670 23223021 PMC3609589

[64] Corbit KC Camporez JPG Tran JL Wilson CG Lowe DA Nordstrom SM Adipocyte JAK2 mediates growth hormone-induced hepatic insulin resistance. JCI Insight. (2017) 2(3):e91001. 10.1172/jci.insight.91001 28194444 PMC5291741

[65] Yang T Qiao S Zhu X. High-dose radiation-resistant lung cancer cells stored many functional lipid drops through JAK2/p-STAT3/FASN pathway. J Cancer Res Clin Oncol. (2023) 149(15):14169–83. 10.1007/s00432-023-05106-1 37553421 PMC11797364

[66] Monterisi S D'Ario G Dama E Rotmensz N Confalonieri S Tordonato C Mining cancer gene expression databases for latent information on intronic microRNAs. Mol Oncol. (2015) 9(2):473–87. 10.1016/j.molonc.2014.10.001 25459350 PMC5528658

[67] Alonso-Montes C Rodríguez-Reguero J Martín M Gómez J Coto E Naves-Díaz M Rare genetic variants in GATA transcription factors in patients with hypertrophic cardiomyopathy. J Investig Med. (2017) 65(5):926–34. 10.1136/jim-2016-000364 28381408

[68] Waters MJ Brooks AJ. JAK2 Activation by growth hormone and other cytokines. Biochem J. (2015) 466(1):1–11. 10.1042/BJ20141293 25656053 PMC4325515

[69] Yang X Jia J Yu Z Duanmu Z He H Chen S Inhibition of JAK2/STAT3/SOCS3 signaling attenuates atherosclerosis in rabbit. BMC Cardiovasc Disord. (2020) 20(1):133. 10.1186/s12872-020-01391-7 32169038 PMC7071770

[70] Li L Zhang Y Qin L. Effect of celecoxib plus standard chemotherapy on cancer prognosis: a systematic review and meta-analysis. Eur J Clin Investig. (2023) 53(6):e13973. 10.1111/eci.13973 36807298

[71] Wei M Lu Z Zhang H Fan X Zhang X Jiang B Aspirin and celecoxib regulate Notch1/Hes1 pathway to prevent pressure overload-induced myocardial hypertrophy. Int Heart J. (2024) 65(3):475–86. 10.1536/ihj.23-614 38825493

